# A new K^+^channel-independent mechanism is involved in the antioxidant effect of XE-991 in an in vitro model of glucose metabolism impairment: implications for Alzheimer’s disease

**DOI:** 10.1038/s41420-022-01187-y

**Published:** 2022-09-20

**Authors:** Silvia Piccirillo, Alessandra Preziuso, Salvatore Amoroso, Tiziano Serfilippi, Francesco Miceli, Simona Magi, Vincenzo Lariccia

**Affiliations:** 1 Department of Biomedical Sciences and Public Health, School of Medicine, University “Politecnica delle Marche”, Via Tronto 10/A, 60126 Ancona, Italy; 2 Department of Neuroscience, University of Naples “Federico II”, Via Pansini 5, 80131 Naples, Italy

**Keywords:** Cellular neuroscience, Alzheimer's disease

## Abstract

Alzheimer’s disease (AD) is a neurodegenerative disorder that represents the first cause of dementia. Although there has been significant progress in AD research, the actual mechanisms underlying this pathology remain largely unknown. There is increasing evidence that oxidative stress, metabolic alterations, and mitochondrial dysfunction are key players in the development and worsening of AD. As a result, in the past few years, remarkable attempts have been made to develop neuroprotective strategies against the impairment of mitochondrial dynamics and cell redox status. In the present study, we reveal a novel antioxidant K^+^ channel-independent effect of the M-current inhibitor XE-991 in SH-SY5Y cells differentiated with retinoic acid (RA) and primary rat cortical neurons exposed to the glycolysis inhibitor glyceraldehyde (GA). This experimental approach aimed to create a condition of hypometabolism accompanied by mitochondrial dysfunction and redox imbalance, as frequently observed in the beginning stage of the disease. We found that XE-991 exerted a neuroprotective action most likely through the resumption of superoxide dismutase (SOD) activity, which was significantly compromised during GA challenge. We also observed that the enhancement of SOD activity was accompanied by a sequence of positive effects; these included the reduction in basal Ca^2+^ levels within cytoplasmic and mitochondrial compartments, the decrease in mitochondrial reactive oxygen species (ROS) production, the modulation of AMPK/mTOR pathway, the recovery of ΔΨ_m_ collapse, the increase in the intracellular ATP content and the decrease in amyloid-β (Aβ) and hyperphosphorylated form of tau protein (pTau) levels. Collectively, our study reveals an off-target antioxidant effect of XE-991 and paves the way toward the further evaluation of new therapeutic uses of already existing molecules to accelerate the process of developing an effective therapy to counteract AD.

## Introduction

Alzheimer’s disease (AD) is a neurodegenerative disorder that represents the first common cause of dementia, affecting approximately 50 million people all over the world [1–3]. The major pathological features of AD are the accumulation of amyloid-β (Aβ) plaques, and intracellular neurofibrillary tangles, which contain the microtubule-associated protein tau in its hyperphosphorylated form (pTau). [3, 4]. Mounting evidence suggests that mitochondrial alteration, metabolic dysfunction, and oxidative damage are upstream events that may cause the subsequent pathogenic cascade typically associated with AD [5, 6]. Several reports have described mitochondrial aberrations in the AD brain [6, 7] and a greater metabolic decline, mainly in regard to the metabolic use of glucose in both the hippocampus and cortex of AD patients in comparison to healthy controls [8]. Moreover, it has been observed that prior to plaque formation, intracellular Aβ may selectively accumulate within mitochondria, thus contributing to bioenergetic impairment [9]. All the above-mentioned events lead to less efficient production of ATP and, at the same time, shift the balance toward pro-oxidant activities [10, 11]. Of note, oxidative stress not only takes part in the onset of AD, but also activates several intracellular pathways that favor the formation of Aβ fragments, thereby promoting the development of the disease [12–14]. Therefore, enhanced oxidative stress could be the trigger, but also the consequence, of the mitochondrial dysfunction characterizing AD [15]. In recent years, remarkable efforts have been made to discover neuroprotective strategies against reactive oxygen species (ROS)-induced damage and mitochondrial impairment [16, 17].

Interestingly, it has been reported that activators of M-current (I_KM_), a subthreshold voltage-gated potassium (K^+^) current encoded by the Kv7/KCNQ channels and critically implicated in the regulation of neuronal excitability in humans [18], exert K^+^ channel-independent neuroprotective actions in different models of excitotoxicity-induced cell damage and cerebral ischemia [19–22]. In particular, it has been observed that retigabine (Ret) and flupirtine, in addition to their role as I_KM_ enhancers, play a neuroprotective role by stimulating the elevation of intracellular glutathione levels [21], thereby counteracting ROS production [19–22], without any involvement of I_KM_-opening activity. Regarding the inhibition of I_KM_, previous studies demonstrated that, in healthy mice, the Kv7 antagonist XE-991 can potentiate learning and memory and revert the impairment of cognitive functions in different neurodegenerative models [23, 24]. However, to date, there is no evidence that inhibitors of the I_KM_ may act through K^+^ channel-independent mechanisms.

Here, we describe a new antioxidant-related K^+^ channel-independent mechanism by which the Kv7 antagonist XE-991 improved cell viability in different in vitro neuronal models exposed to the glycolysis inhibitor glyceraldehyde (GA), which is able to create a condition of hypometabolism accompanied by mitochondrial dysfunction and redox imbalance, thus reproducing an environment frequently observed in the beginning stage of the disease [25, 26].

## Results

### Retigabine and XE-991 exerted a neuroprotective effect in GA-challenged RA-differentiated SH-SY5Y cells

**Fig. 1:**
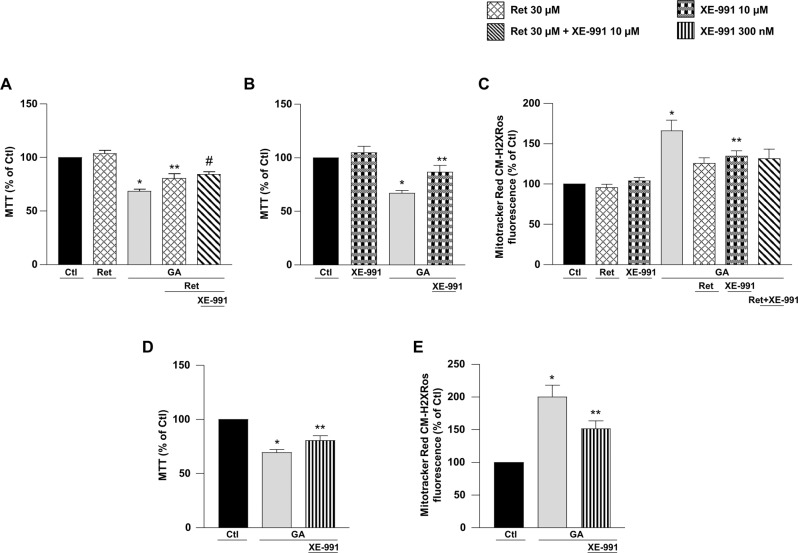
The neuroprotective effect of Ret (30 µM) and XE-991 (300 nM–10 µM) against GA-induced cell damage in RA-differentiated SH-SY5Y cells. **A**, **B**, **D** Effect of Ret and XE-991 on cell viability, assessed by MTT assay, and **C**, **E** mitochondrial ROS production assessed by measuring MitoTracker Red CM-H2XRos fluorescence intensity in cells challenged with GA. Cells were pretreated with Ret (30 µM) and XE-991 (300 nM–10 µM) for 1 h and then exposed to GA (1 mM) for 24 h (without withdrawing Ret and XE-991). In each experiment MTT reduction and the fluorescence intensity of MitoTracker Red CM-H2XRos were expressed as control percentages. Differences among means were evaluated by one-way ANOVA followed by Dunnett’s post hoc test. **A** F (4, 35) = 32.35. Each column depicts the mean ± S.E.M. of at least four independent experiments that were performed in triplicate. *Significant versus all groups (*p* < 0.0001 versus control groups, *p* < 0.001 versus Ret + XE-991 + GA, *p* < 0.01 versus Ret + GA); **significant versus control groups (*p* < 0.0001) and GA (*p* < 0.01); ^#^significant versus Ctl (*p* < 0.0001), Ret and GA (*p* < 0.001). **B** F (3, 18) = 15.52. Each column depicts the mean ± S.E.M. of at least four independent experiments that were performed in triplicate. *Significant versus all groups (*p* < 0.0001 versus control groups, *p* < 0.01 versus XE-991 + GA); **significant versus XE-991 (*p* < 0.05) and GA (*p* < 0.01). **C** F (6, 30) = 8.198. The bar plot shows the mean ± S.E.M. of fluorescence increase evoked by ROS production. For each experimental group, the statistical analysis was performed by using the basal values derived from at least three independent experiments, and 100–150 cells were recorded for each session. *Significant versus all groups (*p* < 0.0001 versus Ctl, *p* < 0.001 versus Ret and XE-991, *p* < 0.05 versus Ret + GA, XE-991 + GA and Ret + XE-991 + GA); **significant versus Ctl and GA (*p* < 0.05). **D** F (2, 21) = 27.57. Each column shows the mean ± S.E.M. of eight independent experiments that were performed in triplicate. *Significant versus all groups (*p* < 0.0001 versus Ctl and *p* < 0.05 versus XE-991 + GA); **significant versus all groups (*p* < 0.001 versus Ctl and *p* < 0.05 versus GA). **E** F (2, 12) = 15.92. The bar plot shows the mean ± S.E.M. of the fluorescence increase evoked by ROS production. For each experimental group, the statistical analysis was performed by using the basal values derived from five independent experiments, and 100–150 cells were recorded for each session. *Significant versus all groups (*p* < 0.001 versus Ctl and *p* < 0.05 versus XE-991 + GA); **significant versus all groups (*p* < 0.05). Ctl control, GA glyceraldehyde, Ret retigabine.

To create an environment reproducing the early stages of AD, we used GA, which is known to alter cell energy metabolism [26] and to induce neurotoxicity through the generation of advanced glycation end products (AGEs) [27–30]. Considering that RA-differentiated SH-SY5Y cells express both the Kv7.2 and Kv7.5 channels (Fig. S1), and the well-known neuroprotective effect of retigabine (Ret), we initially investigated whether the modulation of Kv7 channels could play a role in our experimental setting. RA-differentiated SH-SY5Y were exposed to Ret (30 µM)—at a concentration fully activating the I_KM_ [31]—1 h before and throughout the duration of GA (1 mM) treatment (Fig. 1A, B). As shown in Fig. 1A, Ret significantly improved cell viability (Fig. 1A). To elucidate whether the neuroprotective effects of Ret could rely on I_KM_ activation, we exposed cells to the I_KM_ inhibitor XE-991 (10 µM) together with Ret. Interestingly, we found that XE-991 failed to antagonize Ret-induced neuroprotection (Fig. 1A), suggesting that the I_KM_ was not involved in the neuroprotection induced by Ret. Therefore, we evaluated the effect of XE-991 (10 µM) in the absence of Ret. As reported in Fig. 1B, XE-991 (10 µM) alone was able to mitigate cell damage induced by GA, further supporting that Kv7 channels were not involved in GA-induced neurotoxicity. Since Ret can exert antioxidant activity [19, 20], we explored this possibility in our experimental setting. We found that Ret significantly reduced mitochondrial ROS production in RA-differentiated SH-SY5Y cells (Fig. 1C). Surprisingly, we found that XE-991 was also able to reduce mitochondrial ROS production after GA challenge (Fig. 1C), confirming that Kv7 channels were not implicated in GA-induced neurotoxicity and revealing a new activity of this compound. Additionally, the Icagen’s N-(6-chloropyridin- 3-yl)-3,4-difluorobenzamide (ICA-27243), a Kv7 channel opener not structurally related to Ret [32], was unable to protect from GA-induced cytotoxicity (Fig. S2). To finally rule out the involvement of Kv7 channels, cells were treated with XE-991 at a concentration of 300 nM, which is not active on Kv7 channels (XE-991 IC_50_ ~0.6 µM) [33] (Fig. 1D, E). Interestingly, this concentration of XE-991 was able to improve cell survival (Fig. 1D) and reduce the increase in mitochondrial ROS generation (Fig. 1E). These data suggested that the protection exerted by XE-991 could be due to K^+^ channel-independent mechanisms, most likely relying on the modulation of antioxidant defenses.

### XE-991 exerted a neuroprotective effect against GA toxicity in primary rat cortical neurons

**Fig. 2:**
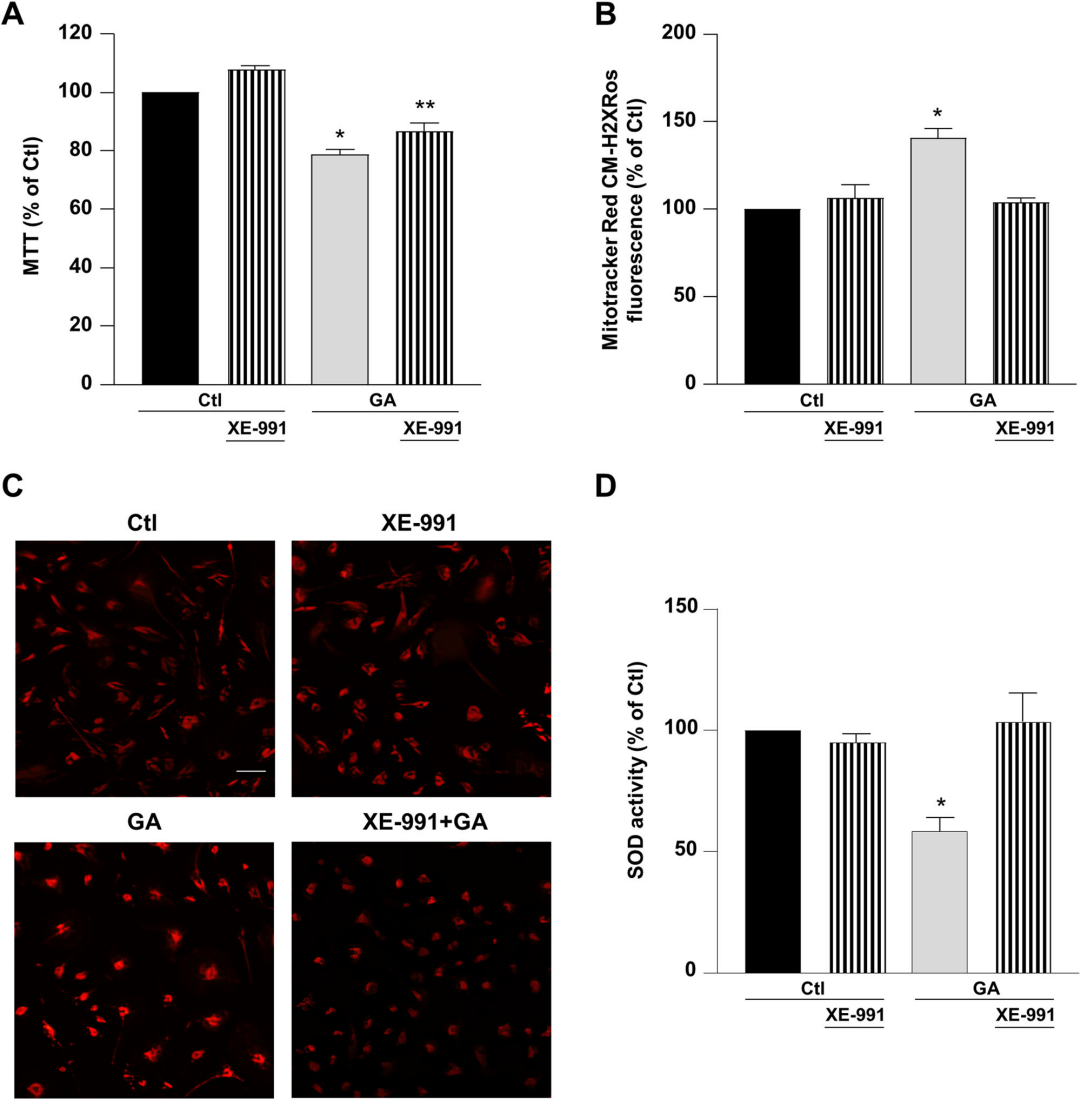
The neuroprotective effect of XE-991 (300 nM) on cell viability, mitochondrial ROS production, and SOD activity measured in primary rat cortical neurons. **A** Evaluation of the effect of 1 h exposure to XE-991 (300 nM) on mitochondrial activity, **B**, **C** mitochondrial ROS production and D) SOD activity. XE-991 (300 nM) was added 1 h before GA (1 mM) exposure and maintained for the whole GA treatment (24 h). In each experiment MTT reduction, the fluorescence intensity of MitoTracker Red CM-H2XRos and SOD activity were expressed as control percentages. Differences among means were evaluated by one-way ANOVA followed by Dunnett’s post hoc test. **A** F (3, 23) = 32.45. Each column depicts the mean ± S.E.M. of at least three independent experiments that were performed in triplicate. *Significant versus all groups (*p* < 0.0001 versus control groups and *p* < 0.05 versus XE-991 + GA); **significant versus all groups (*p* < 0.001 versus Ctl, *p* < 0.0001 versus XE-991 and *p* < 0.05 versus GA). **B** F (3, 11) = 19.02. The bar plot shows the mean ± S.E.M. of the fluorescence increase evoked by ROS production. For each experimental group, the statistical analysis was performed by using the basal values derived from at least three independent experiments, and 100–150 cells were recorded for each session. *Significant versus all groups (*p* < 0.0001). **C** Representative pictures of mitochondrial ROS obtained by MitoTracker Red CM-H2XRos staining deriving from at least three independent experiments. Scale bar = 50 µm. **D** F (3, 18) = 8.763. Each column shows the mean ± S.E.M. of at least four independent experiments that were performed in triplicate. *Significant versus all groups (*p* < 0.01 versus Ctl, *p* < 0.05 versus XE-991, and *p* < 0.001 versus XE-991 + GA).

Once it was demonstrated that XE-991 could act through Kv7 channel-independent mechanisms in RA-differentiated SH-SY5Y cells, we focused on investigating whether XE-991 (300 nM) exposure could mitigate cell damage induced by GA in primary rat cortical neurons. As shown in Fig. 2, similar findings were observed in rat primary cortical neurons, where XE-991 significantly ameliorated cell viability (Fig. 2A) and reduced the rise of mitochondrial ROS production (Fig. 2B, C).

### XE-991 stimulated superoxide dismutase (SOD) activity

Considering the lack of evidence that inhibitors of the I_KM_ may act through K^+^ channel-independent mechanisms, we further explored the mechanism underlying XE-991 antioxidant activity. We focused our attention on superoxide dismutase (SOD) enzymes, which are essential for neuronal viability and defense against oxidative stress [34]. For instance, in the mouse model Tg2576 AD, SOD deficiency promotes the appearance of an AD-like phenotype, including the aggregation of Aβ peptide and the hyperphosphorylation of tau [35]. Further studies conducted in the same model demonstrated that the overexpression of SOD prevents memory impairment and amyloid plaque deposition [36]. Therefore, we sought to explore whether XE-991 could have a regulatory effect on these antioxidant enzymes in primary rat cortical neurons exposed to GA. The results showed that SOD activity was significantly reduced in primary rat cortical neurons treated with GA. Interestingly, when neurons were treated with XE-991, SOD activity was fully restored (Fig. 2D). The same result was observed in RA-differentiated SH-SY5Y, where 1 h exposure of XE-991 totally rescued the reduction in SOD activity induced by GA (Fig. S3).

### XE-991 reversed the elevation of Ca^2+^ levels induced by GA within cytoplasmic and mitochondrial compartments in primary rat cortical neurons

**Fig. 3:**
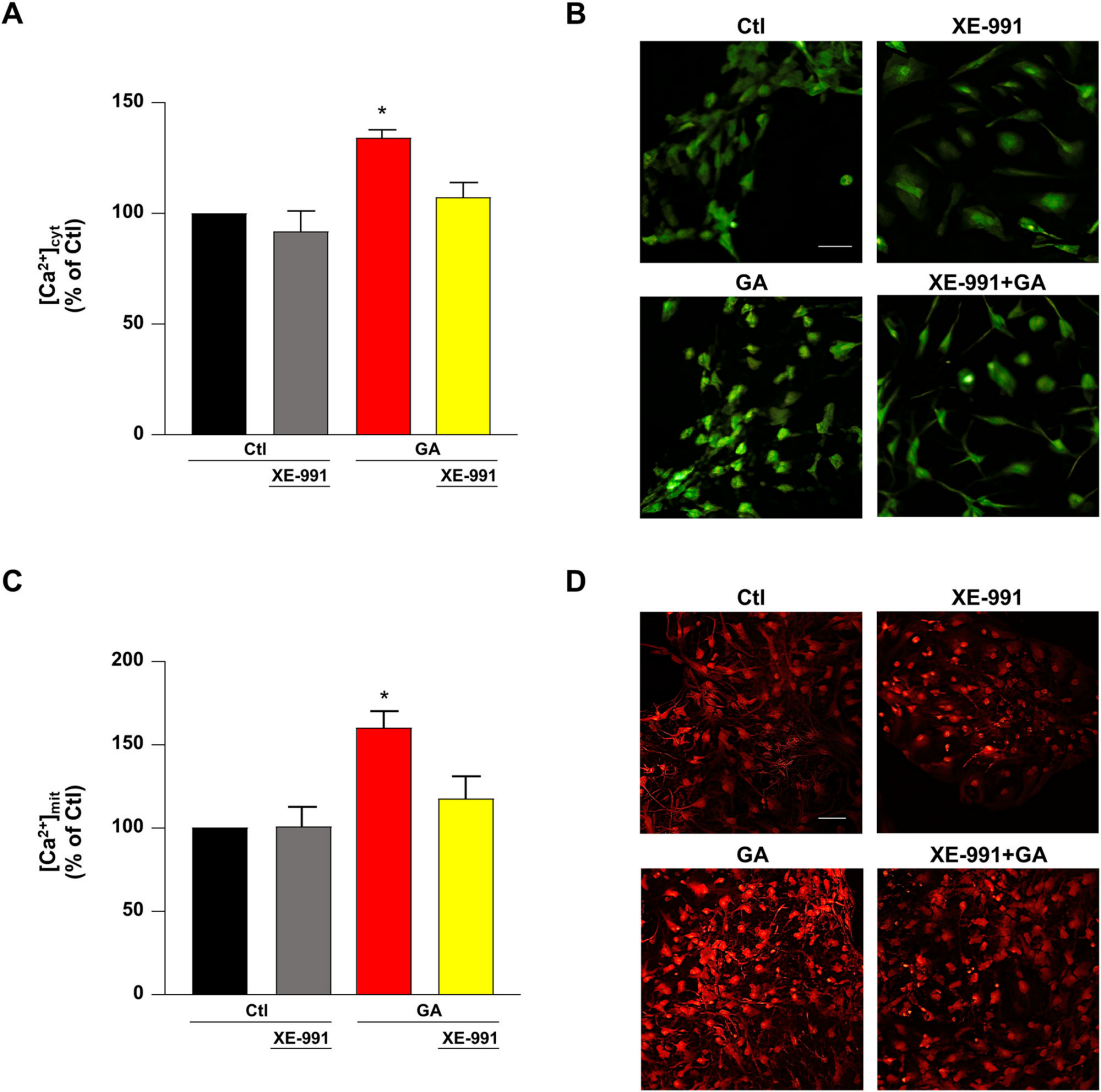
Evaluation of the effect of XE-991 on the increase in cytoplasmic and mitochondrial Ca^2+^ levels induced by GA in primary rat cortical neurons. **A**, **C** The bar plots depict average data of the cytoplasmic (**A**) and mitochondrial (**C**) basal Ca^2+^ levels. The fluorescence intensity is transformed into calibrated [Ca^2+^] by applying the following equation: [Ca^2+^] = Kd (F − F_min_) / (F_max_ − F) where Kd is the Ca^2+^ dissociation constant of the indicator (Kd Fluo-4-AM = 345 nM; Kd Rhod-2-AM = 570 nM); F_min_ and F_max_ represent the intensities of fluorescence at zero and saturating [Ca^2+^], respectively; and F represents the intensity of fluorescence at any given time. In each experiment, [Ca^2+^] was expressed as control percentage. **B**, **D** Representative images of the basal Ca^2+^ levels within cytoplasm (**B**) and mitochondria (**D**). Scale bar 50 µm. Differences were evaluated by one-way ANOVA followed by Dunnett’s post hoc test. **A** F (3, 10) = 11.67. For each experimental group, the statistical analysis was performed by using the basal values derived from three independent experiments, and 100–150 cells were recorded for each session. *Significant versus all groups (*p* < 0.01 versus Ctl, *p* < 0.001 versus XE-991, and *p* < 0.05 versus XE-991 + GA). **C** F (3, 15) = 8.091. For each experimental group, the statistical analysis was performed by using the basal values derived from at least four independent experiments, and 100–150 cells were recorded for each session. *Significant versus all groups (*p* < 0.01 versus control groups and *p* < 0.05 versus XE-991 + GA).

The alteration of Ca^2+^ handling is a central driver of AD progression linking amyloid metabolism to neurodegeneration [7, 37]. Since our previous experiments demonstrated that the metabolic perturbation elicited by GA was accompanied by the alteration of the mechanisms underlying Ca^2+^ homeostasis [25], we evaluated whether the protection exerted by XE-991 could also reflect regulation of Ca^2+^ balance. To this aim, we measured both cytoplasmic and mitochondrial Ca^2+^ levels. In Fluo-4-AM and Rhod-2-AM loaded rat cortical neurons, we found that exposure to GA significantly increased basal Ca^2+^ levels in both cytoplasmic (Fig. 3A, B) and mitochondrial compartments (Fig. 3C, D), whereas XE-991 reversed the elevation of both cytoplasmic and mitochondrial Ca^2+^ (Fig. 3).

### XE-991 rescued the inner mitochondrial membrane depolarization and ATP reduction induced by GA challenge in primary rat cortical neurons

**Fig. 4:**
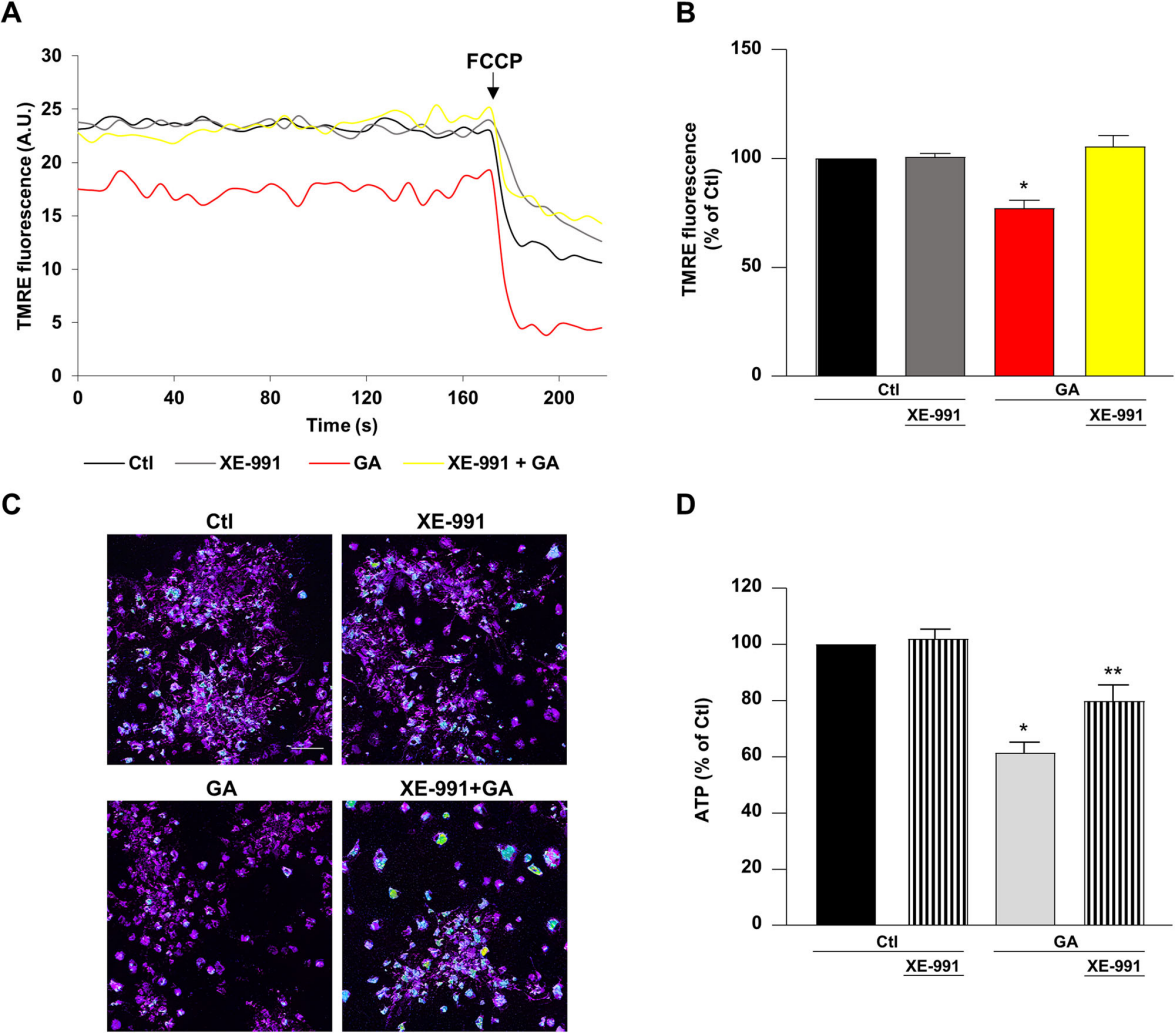
Effect of XE-991 on mitochondrial membrane potential and ATP production in GA challenged primary rat cortical neurons. **A** Representative records showing mitochondrial membrane potential measurements under different experimental conditions, namely control (black line), exposure to XE-991 (gray line), 24 h of GA challenge (red line), and 24 h of GA challenge in the presence of XE-991 (yellow line). **B** Quantification of mitochondrial membrane potential and **D** ATP production under different experimental conditions. **C** Representative pictures of mitochondrial membrane potential measurements. Scale bar 50 µm. In each experiment intracellular TMRE fluorescence and ATP levels were reported as control percentages. Differences among means were evaluated by one-way ANOVA followed by Dunnett’s post hoc test. **A**, **B** After permeabilizing cells with digitonin (5 µM), mitochondrial membrane potential was evaluated by using a nonquenching concentration (10 nM) of the inner mitochondrial membrane potential indicator TMRE. FCCP (20 µM) was added at the end of the recording session as internal control. **B** F (3, 12) = 15.57. Each column depicts the mean ± S.E.M. of 50–100 cells recorded in four different sessions. *Significant versus all groups (*p* < 0.001). **D** F (3, 37) = 24.03. Each column depicts the mean ± S.E.M. of at least four independent experiments that were performed in triplicate. *Significant versus all groups (*p* < 0.0001 versus control groups and *p* < 0.01 versus XE-991 + GA); **significant versus all groups (*p* < 0.001 versus Ctl, *p* < 0.05 versus XE-991, and *p* < 0.01 versus GA).

Mitochondrial energy production, through oxidative phosphorylation, generates an inner membrane potential (ΔΨ_m_), which represents the main driving force for ATP synthesis [38]. We have previously observed that in primary rat cortical neurons a ΔΨ_m_ loss occurred in parallel with the intracellular ATP depletion induced by GA [26]. Therefore, we first evaluated whether XE-991 could influence ΔΨ_m_ collapse. When cells were pretreated with XE-991, the reduction in ΔΨ_m_ induced by GA exposure was completely prevented (Fig. 4A–C). Given that XE-991 counteracted the ΔΨ_m_ collapse in the background of GA challenge, we investigated whether XE-991 exposure could also influence the content of intracellular ATP (Fig. 4D). Our data showed that pretreatment with XE-991 completely rescued the intracellular ATP reduction induced by GA challenge (Fig. 4D).

### XE-991 counteracted the rise of AD biomarker levels induced by GA in primary rat cortical neurons and RA-differentiated SH-SY5Y cells

**Fig. 5:**
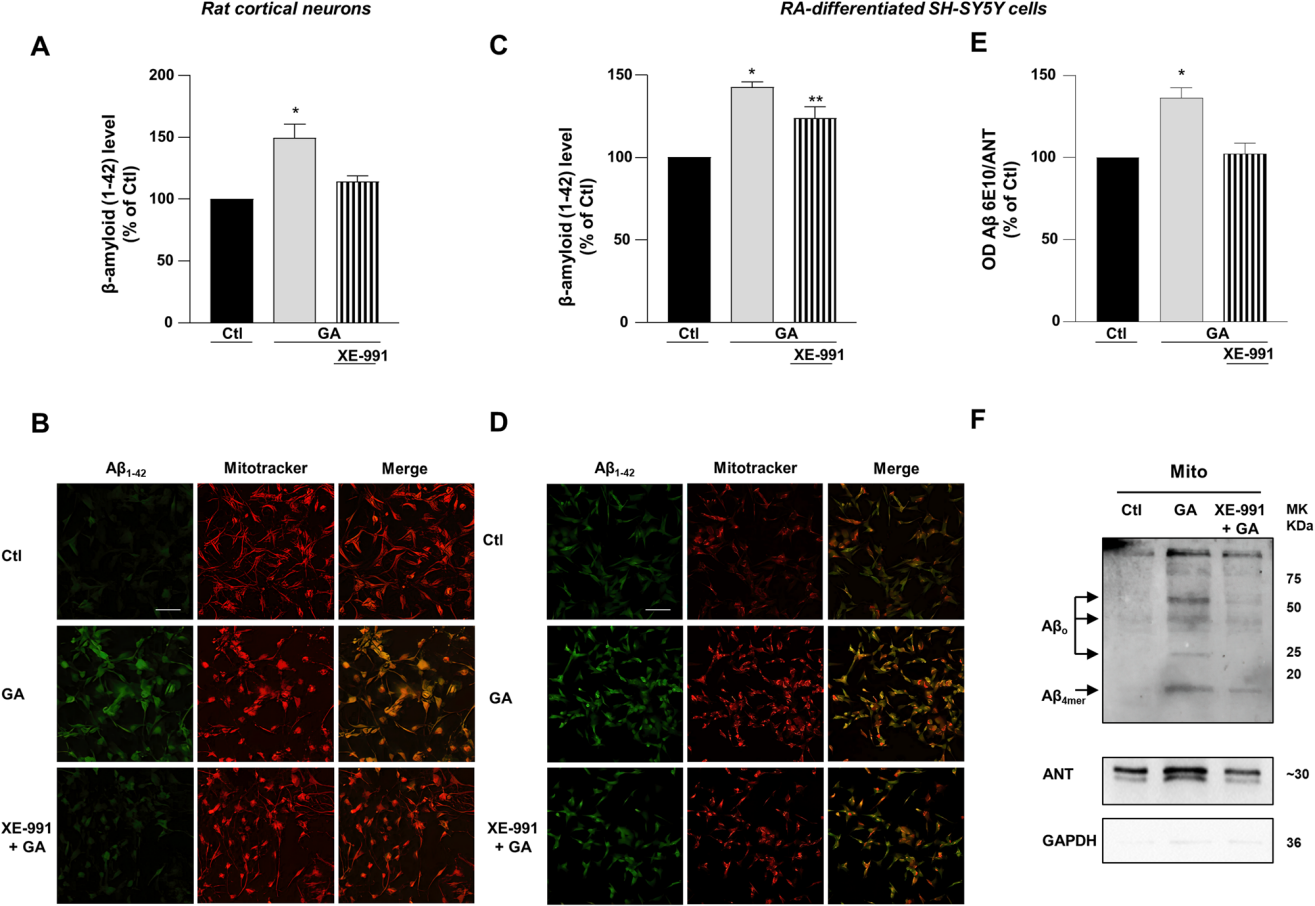
Aβ expression in primary rat cortical neurons and RA-differentiated SH-SY5Y cells treated with GA and exposed to XE-991. **A**, **C**, **E** Quantification and **B**, **D**, **F** representative pictures of Aβ expression. The protein Aβ was detected by immunofluorescence staining (**A**–**D**) and western blot (**E**, **F**). **F** Immunoblotting analysis using the 6E10 antibody showed various sizes of bands corresponding to Aβ aggregates/oligomers (Aβ tetramers (Aβ_4mer_) ~17, Aβ oligomers (Aβ_o_) ~25, ~40, and >~50 kDa). ANT was used as loading control to verify mitochondrial isolation, and GAPDH was used as cytoplasmic marker. In each experiment both the intensity of fluorescence and normalized optical density values of Aβ were reported as control percentages. Scale bar 50 µM. Differences among means were evaluated by one-way ANOVA followed by Dunnett’s post hoc test. **A** F (2, 6) = 12.53. Each column depicts the mean ± S.E.M. of three independent experiments (50–100 cells for each experimental group were analyzed). *Significant versus all groups (*p* < 0.01 versus Ctl, *p* < 0.05 versus XE-991 + GA). **C** F (2, 15) = 21.49. Each column depicts the mean ± S.E.M. of six independent experiments (for each experimental group, 50–100 cells were analyzed). *Significant versus Ctl (*p* < 0.0001) and XE-991 + GA (*p* < 0.05); **significant versus Ctl (*p* < 0.01) and GA (*p* < 0.05). **E** F (2, 9) = 14.99. Each column depicts the mean ± S.E.M. of four independent experiments. *Significant versus all groups (*p* < 0.01).

**Fig. 6:**
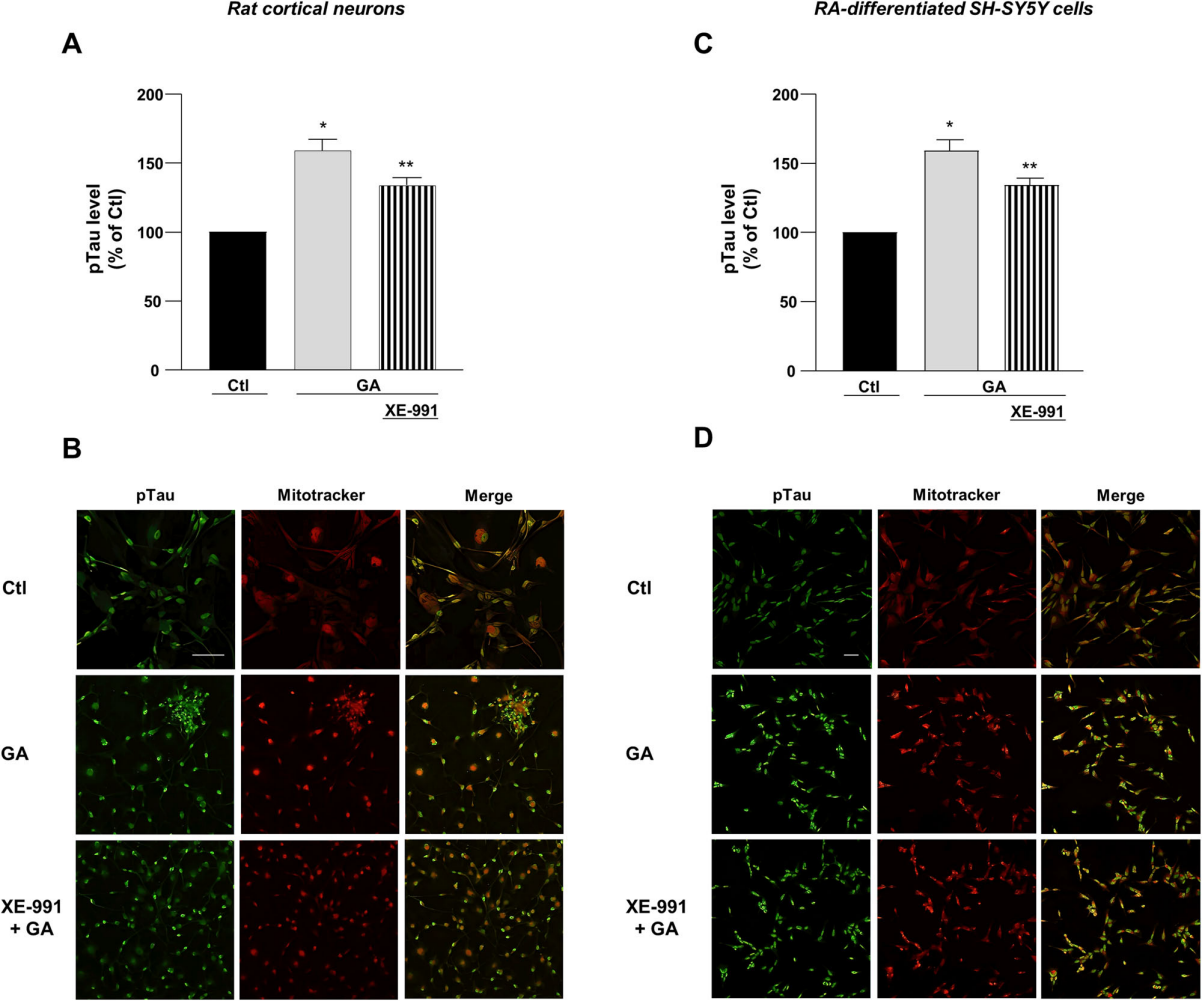
pTau expression in primary rat cortical neurons and RA-differentiated SH-SY5Y cells treated with GA and exposed to XE-991. **A**, **C** Quantification and **B**, **D** representative pictures of pTau expression. pTau was identified by immunofluorescence staining (**A**–**D**). Scale bar 50 µM. The intensity of fluorescence of pTau was reported as control percentage. Differences among means were evaluated by one-way ANOVA followed by Dunnett’s post hoc test. **A** F (2, 6) = 26.96. Each column depicts the mean ± S.E.M. of independent experiments (for each experimental group, 50–100 cells were analyzed). *Significant versus all groups (*p* < 0.0001 versus Ctl, *p* < 0.01 versus XE-991 + GA); **significant versus all groups (*p* < 0.01). **C** F (2, 12) = 43.43. Each column depicts the mean ± S.E.M. of five independent experiments (for each experimental group, 50–100 cells were analyzed). *Significant versus Ctl (*p* < 0.0001) and XE-991 + GA (*p* < 0.01); **significant versus Ctl (*p* < 0.001) and GA (*p* < 0.01).

In AD, neuronal loss correlates with pathological burdens of Aβ plaques deposition and intracellular neurofibrillary tangles composed of pTau fibrils [39]. As previously reported, cells treated with GA showed a marked increase in both extracellular Aβ_1–42_ and intracellular pTau levels [25], therefore, we next wondered whether the neuroprotective effects of XE-991 would also extend to AD biomarker levels. Interestingly, we found that in both primary rat cortical neurons and RA-differentiated SH-SY5Y treated with XE-991, the cellular accumulation of both Aβ (Fig. 5) and pTau (Fig. 6) was significantly reduced compared to what was observed in GA-challenged cells. Moreover, immunofluorescence analysis showed the colocalization of intracellular Aβ with mitochondria (Fig. 5B, D). To further assess the deposition of Aβ in the mitochondria, we isolated the mitochondrial fraction from RA-differentiated SH-SY5Y cells and assessed Aβ levels by western blot. As reported in Fig. 5E, F (Fig. S5), in our mitochondrial preparations, we were able to detect a range of Aβ 6E10-immunoreactive bands corresponding to oligomers and aggregates, whose levels were higher after GA treatment, confirming the ability of GA to increase Aβ deposition and the potential of XE-991 to halt this effect.

### XE-991 ameliorated cell viability through the modulation of AMP-activated protein kinase (AMPK)- mammalian target of rapamycin (mTOR) signaling pathway

**Fig. 7:**
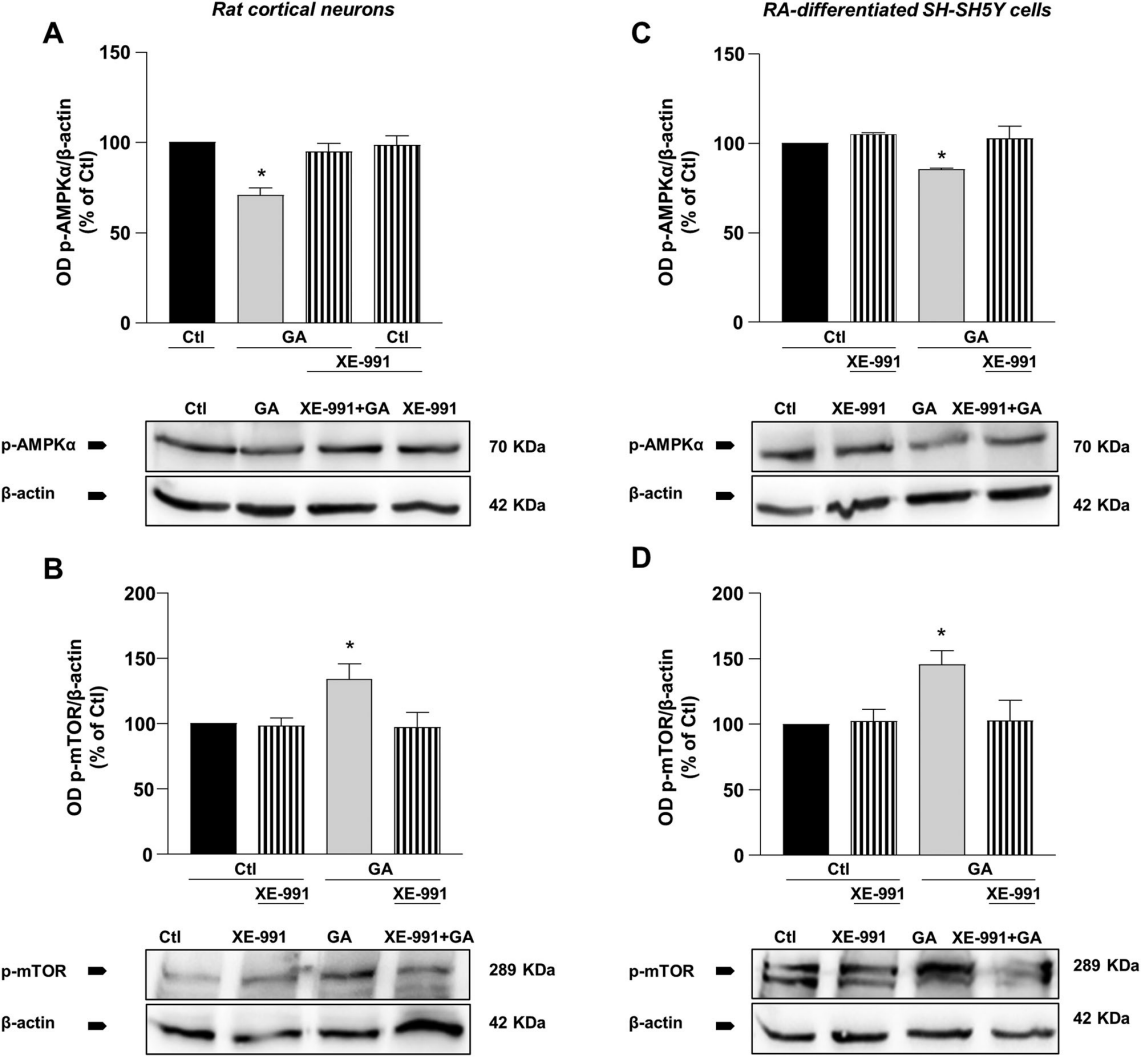
Effect of XE-991 on AMPK/mTOR pathway in primary rat cortical neurons and RA differentiatedSH-SY5Y cells challenged with GA. Quantification of (**A**, **C**) p-AMPKα and (**B**, **D**) p-mTOR expression in primary rat cortical neurons (**A**, **B**) and RA-differentiated SH-SY5Y (**C**, **D**) treated with GA and exposed to XE-991. As loading control, β-actin was used. Normalized optical density values were reported as control percentages. Differences among means were evaluated by one-way ANOVA followed by Dunnett’s post hoc test. **A** F (3, 12) = 11.47. Each column depicts the mean ± S.E.M. of four independent experiments. *Significant versus all groups (*p* < 0.001 versus Ctl; *p* < 0.01 versus XE-991 and XE-991 + GA). **B** F (3, 12) = 4.096. Each column depicts the mean ± S.E.M. of four independent experiments. *Significant versus all groups (*p* < 0.05). **C** F (3, 11) = 5.592. Each column depicts the mean ± S.E.M. of at least three independent experiments. *Significant versus all groups (*p* < 0.05). **D** F (3, 12) = 4.903. Each column depicts the mean ± S.E.M. of at least three independent experiments. *Significant versus all groups (*p* < 0.05).

To go further into the mechanisms by which XE-991 promotes cell survival, we explored potential signaling pathways implicated in GA-induced neurotoxicity and assessed the effect of XE-991. In particular, previous reports demonstrated that oxidative stress stimuli (including AGEs formation) and AMPK inactivation are strictly connected [40, 41], suggesting a role of redox balance in modulating AMPK and in committing the cell to death under conditions of glucose metabolism impairment. In this view, we identified AMPK as a possible complex that could be influenced by GA treatment. Consistently, GA significantly reduced the levels of phosphorylated (Thr172) AMPKα (p-AMPKα) in both rat cortical neurons and RA-differentiated SH-SY5Y cells (Figs. 7A, C and Fig. S6). As a downstream target of AMPK, we then checked for mTOR, which is known to be deregulated in AD [42, 43]. We observed that GA significantly increased phosphorylated (Ser2448) mTOR expression (p-mTOR) (Fig. 7B, D and Fig. S7). Interestingly, XE-991 treatment reversed the reduction in p-AMPKα and the increase in p-mTOR expressions observed after GA treatment (Fig. 7).

## Discussion

In the current study, we revealed a novel antioxidant K^+^ channel-independent effect of the I_KM_ inhibitor XE-991. In particular, by using GA, we created a condition of hypometabolism accompanied by mitochondrial dysfunction and redox imbalance [25, 26], thus reproducing an environment that often characterizes the early stages of AD. In this setting, we provided evidence that, in both RA-differentiated SH-SY5Y and primary rat cortical neurons, XE-991 exerted a protective action, most likely through the resumption of SOD activity, which was significantly compromised during GA challenge. These results are in line with previously reported studies showing that other drugs targeting neuronal Kv7 channels (Kv7.2–7.5), such as retigabine, act as free radical scavengers without any involvement of I_KM_ [19–22, 44]. Of note, we determined that the increase in SOD activity induced by XE-991 occurred in parallel with other major findings, namely, (1) the reduction in the basal Ca^2+^ levels within cytoplasmic and mitochondrial compartments, (2) the decrease in mitochondrial ROS production, (3) the recovery of ΔΨ_m_ collapse, (4) the restoration of the intracellular ATP content, and (5) the decrease in Aβ and pTau levels. We also checked for important death paradigms that could be potentially involved in GA-induced neurotoxicity and found the alteration of the AMPK-mTOR signaling pathway, which was restored by XE-991. These protective effects of XE-991 were observable at a lower concentration than that required to inhibit Kv7 channels, ruling out their involvement. Although our results may warrant further exploration, they support the role of oxidative stress as a driving force contributing to neurodegeneration [38], as reported in several studies, demonstrating that the restoration of antioxidant defenses improves neuronal viability, alleviates neurological dysfunctions, and reduces the risk of developing AD [35, 45–50]. Therefore, oxidative stress may be a promising target for therapeutic interventions.

**Fig. 8:**
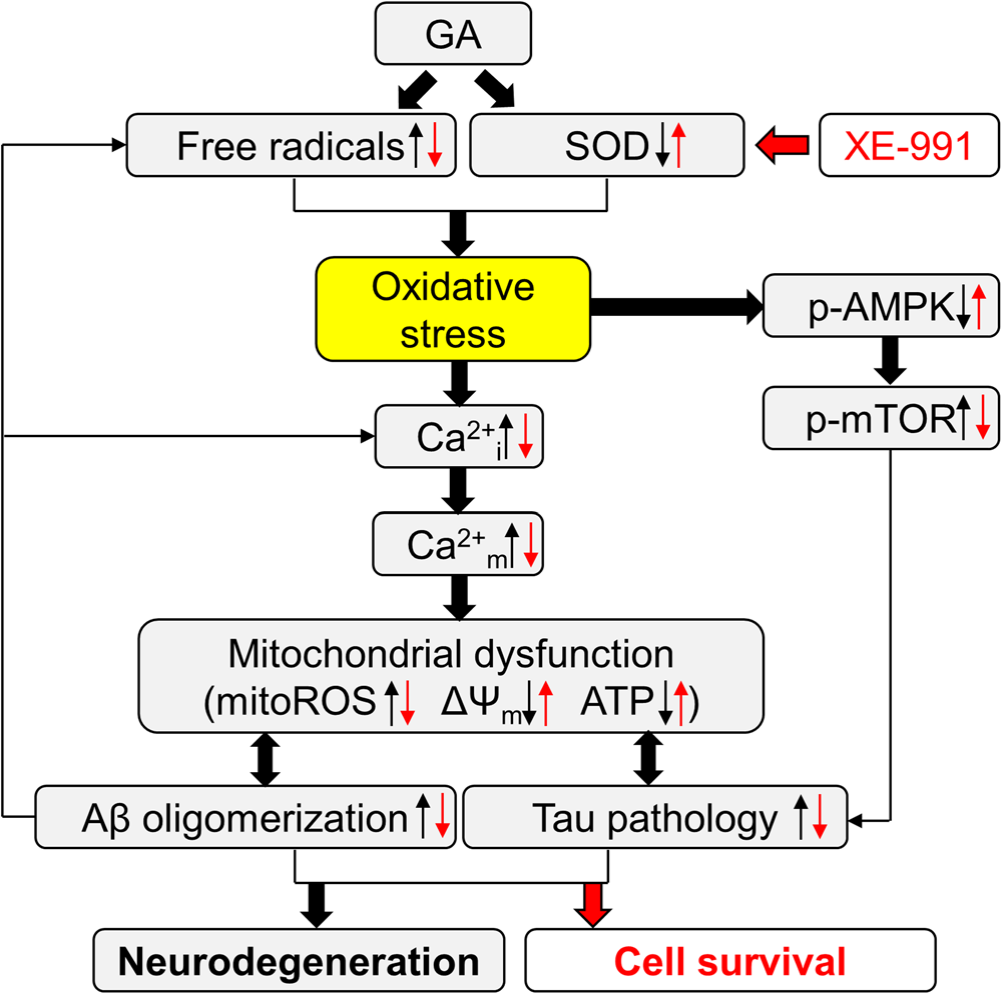
Working hypothesis showing: (1) how oxidative stress may represent the main driving force contributing to the subsequent complex AD pathogenic cascade, and (2) how the restoration of SOD activity induced by XE-991 may halt this cascade.

We have previously demonstrated that the metabolic impairment induced by GA is accompanied by a perturbation of the overall oxidative status of neuronal cells [25]. In the present study, we also found that GA dramatically reduced SOD activity, supporting the neuronal shift to a pro-oxidant status. In agreement with this finding, in APP transgenic mice and in the postmortem frontal cortex of AD patients, it has been shown that the impairment of SOD activity occurs concomitantly with the increase in Aβ levels [45, 51]. In the literature, significant evidence indicates that oxidative status may affect the activity of a variety of systems involved in controlling Ca^2+^ homeostasis, likely inducing dysregulation of intracellular Ca^2+^ levels [52]. Consistent with this finding, we showed that GA challenge was accompanied by a dramatic rise in intracellular Ca^2+^ levels, which concomitantly affected Ca^2+^ homeostasis within mitochondria as a consequence of their buffering activity. Overall, we propose that this phenomenon may represent the trigger for a cascade of events precipitating mitochondrial function and leading to neuronal death. Increased levels of Ca^2+^ within mitochondria could be responsible for the observed collapse of the ΔΨ_m_ and, at the same time, might augment the imbalance between pro-oxidant and antioxidant defenses, thus inducing the formation of mitochondrial ROS. Consequently, we can speculate that ATP-production systems underwent dramatic changes, as supported by the observed drop in intracellular ATP content. The significant reduction in neuronal viability occurring in this experimental setting represented a downstream event, which might have been further augmented by the increase in both Aβ and pTau levels. In fact, several reports indicate that oxidative stress and Ca^2+^ dysregulation may both converge on the abnormal production and deposition of Aβ peptide [53, 54], and on Tau phosphorylation [7, 55]. For instance, the rise of the mRNA and activity of β and γ secretases—which provide consecutive cleavages of Amyloid Precursor Protein (APP) [56], thus generating toxic Aβ peptides—correlates with the increase in oxidative biomarker (e.g., hydroxynonenal (HNE) and H_2_O_2_) levels [57]. Moreover, in line with these observations and with the experimental model proposed here, it has been shown that AGEs deriving from GA can augment the levels of APP and Aβ through the formation of ROS [27]. The alteration of Ca^2+^ homeostasis, which can occur in parallel with the imbalance of redox status [7], could contribute as well to alter both Aβ and pTau levels. In this regard, there is evidence that Ca^2+^ may directly interact with β-secretase, therefore enhancing its proteolytic activity and exacerbating Aβ formation [58]. In addition, a sustained increase in intracellular Ca^2+^ activates many Ca^2+^-sensitive proteins, including key tau kinases (e.g., glycogen synthase kinase 3β and cyclin-dependent kinase 5), which promote Tau phosphorylation, and calpains, which regulate the cleavage of APP [59]. On the other hand, Aβ and pTau deposition can themselves be a trigger for oxidative stress and both cytosolic and mitochondrial Ca^2+^ deregulation [60, 61]. In our experimental setting, we provided evidence for a direct accumulation of Aβ within mitochondria, which is suggestive of pathological crosstalk between Aβ accumulation, oxidative damage, and Ca^2+^ dysregulation that incites neuronal injury, which is in line with the complex and multifactorial nature of AD pathology that cannot be simply tracked to single unipolar mechanism [62]. To gain further insights into our experimental model, we also attempted to check the involvement of death paradigms typically associated with AD, and we focused on the possible involvement of AMPK [42]. AMPK complex is primarily known as a sensor of the intracellular metabolism, even though AMPK activity can be also modulated by the intracellular redox balance [40, 63, 64]. Accordingly, neuronal samples with decreased AMPK activation exhibit alteration of mitochondrial dynamics and oxidative damage [65–67], two major AD features that were recapitulated by our experimental model. A decrease in AMPK activity has been observed in AD pathology and aging [68]. Here we found a significant reduction in AMPK activity in cells exposed to GA, which is known to alter energy metabolism and augment oxidative stress through the formation of AGEs [27–30]. In line with this finding, previous studies have described the neuroprotective role of AMPK activation against AGEs-induced oxidative stress and mitochondrial dysfunction [69]. Since AMPK regulates mTOR activity, and both have been shown to be deregulated in neurodegenerative diseases [42, 43, 68, 70, 71], we also checked for mTOR activation. As similarly seen in a different cell model exposed to AGE [41], downregulation of p-AMPKα expression was accompanied by an upregulation of p-mTOR expression, and both these changes were reversed by XE-991. Overall, these findings highlighted the potential of XE-991 to target metabolic dysfunctions that are the characteristic signatures of AD pathology. Although the exact role of the crosstalk between AMPK and mTOR pathway in AD is still debated, there is consensus on the inhibitory effect of mTOR on autophagy [43], a process that in AD has been linked to the increase in misfolded aggregated proteins and injured organelles, with main regard to mitochondria [72]. A possible speculation is that the demonstrated ROS scavenging activity of XE-991 may create a favorable environment for the activation of AMPK, which in turn may inhibit mTOR activity, finally increasing autophagy induction and, therefore, facilitating the clearance of the aberrant aggregates of Aβ and tau proteins. In Fig. 8, we illustrate our working hypothesis showing how metabolic impairment (here generated by GA) may represent the upstream event favoring the imbalance of cell oxidative status, the inactivation of AMPK, and the activation of mTOR, triggering a vicious self-feeding cycle culminating in neurodegeneration (Fig. 8).

Taken together, this study demonstrated that XE-991 could exert an antioxidant effect based on a K^+^ channel-independent mechanism, which possibly involves the enhancement of SOD activity. Our results confirm that oxidative imbalance is an initial event in AD and that it could trigger a complex neurodegenerative cascade. More importantly, we believe that our study may pave the way toward the further evaluation of new therapeutic uses of already existing molecules, to accelerate the process of developing an effective therapy to counteract AD.

## Materials and methods

### Cell culture and treatment

The SH-SY5Y human neuroblastoma cell line, purchased from American Type Culture Collection (CRL-2266), was cultured as a monolayer in polystyrene dishes (100 mm diameter) in Dulbecco’s Modified Eagle’s Medium (Corning, New York, USA) supplemented with 10% fetal bovine serum (FBS), 100 U/ml penicillin, and 100 μg/ml streptomycin (Corning). Cells were grown in a humidified incubator at 37 °C and 5% CO_2_, renewing the culture medium every 48 h. To induce neuronal differentiation, SH-SY5Y cells were exposed to 10 µM all-trans retinoic acid (RA) for 6 days [73]. Cortical neurons were isolated from the cortex of Wistar rat pups (P2–P4) (Cat. 003WISTAR, Charles River, Lecco, Italy) as previously described [25]. The use of animals and procedures were in full compliance with the Ethics Committee for Animal Experiments of the University “Politecnica delle Marche” and in strict accordance with the guidelines of the Italian Ministry of Health (D.L. 26/2014). In brief, cortices were placed in ice-cold PBS soon after isolation and then trypsinized (0.05% trypsin/EDTA) for 15 min at 37 °C. After an additional step of homogenization, neurons were obtained. To assess mitochondrial activity and intracellular ATP content, neurons were grown on poly-D-lysine-coated glass coverslips in a 12 multiwell cell culture plate (6 × 10^5^ cells/well). For live imaging and western blot analysis, neurons were grown on poly-D-lysine-coated glass coverslips in a 6 multiwell cell culture plate (1.3 × 10^6^ cells/well). Neuronal cultures were maintained in Neurobasal medium (Gibco-Invitrogen, Paisley, UK) supplemented with B27 (Gibco-Invitrogen) and 2 mM glutamine in the presence of penicillin/streptomycin and cultured at 37 °C in a humidified atmosphere of 5% CO_2_. The medium was changed twice a week, and experiments were performed between 10 and 14 DIV (days in vitro). To induce an AD-like phenotype, cells were treated with GA for 24 h [25]; Ret (30 µM), XE-991 (300 nM–10 µM) and/or ICA-27243 (30 µM) were added to the culture medium 1 h before GA treatment and kept in contact with the cells throughout the whole period of GA exposure. To analyze cytoplasmic and mitochondrial basal Ca^2+^ levels, neurons were pretreated with XE-991 for 1 h followed by GA exposure for 16 h. This timeframe of GA exposure was chosen since we have already demonstrated that, at this time, cell viability starts to be significantly compromised, and systems controlling Ca^2+^ homeostasis are also dysregulated [25].

### Mitochondrial activity

The MTT assay was used to evaluate the metabolic activity and viability of cells. This test is based on the ability of viable cells to reduce the MTT [3-(4,5-dimethylthiazol-2-yl)-2,5-diphenyltetrazolium bromide] into insoluble formazan by the activity of the mitochondrial NADH-dependent oxidoreductase enzymes [25]. Briefly, cells cultured in 12 multiwell plates were treated according to the assigned group and, at the end of the treatment, incubated for 1 h with 1 ml of MTT reagent (dissolved in PBS at 0.5 mg/ml) in the dark at 37 °C and in a 5% CO_2_ atmosphere in a humidified incubator. The supernatant was then removed and centrifuged at 12,000 × *g* for 5 min to collect formazan crystals that were finally dissolved in 1 ml of DMSO. The absorbance was read at 540 nm by using a Victor Multilabel Counter plate reader (Perkin Elmer, Waltham, MA, USA). Cell viability was expressed as a percentage of the control value.

### Evaluation of mitochondrial ROS production

ROS production from mitochondria was visualized using the mitochondrial-targeted dye MitoTracker CM-H2XRos [74] (Invitrogen Life Technologies, Carlsbad, CA, USA). Cells grown on glass coverslips (coated with poly-D-lysine for neuronal cultures) were treated according to the protocol routines and then loaded with 300 nM of the dye for 30 min, at 37 °C, in the dark. CM-H2XRos was excited at 561 nm, and fluorescence emission was collected above 580 nm and recorded for ~60 s using a confocal microscope Zeiss 510 LSM (Carl Zeiss, Milan, Italy). Changes in the red fluorescence were analyzed offline after image acquisition. Fluorescence values were reported as percentages of the control value.

### Measurement of SOD activity

SOD activity was determined using the Superoxide Dismutase Assay kit (Cayman Chemical Co., Inc., Ann. Arbor, MI) according to instructions provided by the manufacturer. Briefly, rat primary cortical neurons were treated as per the assigned experimental group, then homogenized in cold buffer [20 mM HEPES buffer (pH 7.2), 1 mM EGTA, 210 mM mannitol, 70 mM sucrose] and finally centrifuged at 1500 × *g* for 5 min at 4 °C. Total SOD activity was measured from the collected supernatants and expressed as a percentage of the control value.

### Evaluation of cytoplasmic and mitochondrial Ca^2+^ levels

Basal Ca^2+^ levels within cytoplasmic and mitochondrial compartments were measured by single-cell computer-assisted videoimaging using a LSM 510 confocal system (Carl Zeiss). In brief, rat primary cortical neurons grown on poly-D-lysine-coated glass coverslips were exposed to 1 h XE-991 followed by 16 h of GA treatment. Then, neurons were loaded with 4 µM Fluo-4-AM (Invitrogen Life Technologies) or 5 µM Rhod-2-AM (Abcam, Cambridge, UK) in the medium for 45 min at 37 °C. After the loading period, neurons were washed in standard buffer solution for an additional 10 min. Finally, neurons were placed into a perfusion chamber mounted onto the stage of an inverted Zeiss Axiovert 200 microscope and perfused with standard buffer solution (in mM: 140 NaCl, 5 KCl, 1 CaCl_2_, 0.5 MgCl_2_, 10 HEPES, 5.5 glucose, buffered to pH 7.4 with NaOH) for about 200 s, at 37 °C using a heated microscope stage and climate box from PeCon GmbH. After 200 s, the standard buffer solution was replaced with calibration solution 1 (EGTA 0 Ca^2+^) until the lowest fluorescent value was stably reached (about 100 s); then, calibration solution 1 was replaced with calibration solution 2 (high Ca^2+^) to obtain the highest fluorescent value (Fig. S4). Solutions were delivered by a peristaltic pump. Images were acquired every 5 s; Fluo-4-AM was excited at 488 nm, and the emitted fluorescence was recorded at 505–530 nm, while Rhod-2- AM was excited at 543 nm, and fluorescence emission was measured from 560 to 600 nm. Fluorescence analysis was performed offline after image acquisition.

Calibration solutions were prepared as previously described with minor modifications [75]. Specifically: calibration solution 1 (EGTA 0 Ca^2+^) contained (in mM) 140 NaCl, 5 KCl, 3 EGTA, 0.5 MgCl_2_, 10 HEPES, 5.5 glucose, 0.01 A23187 pH 7.4; calibration solution 2 (high Ca^2+^) contained (in mM) 140 NaCl, 5 KCl, 4 CaCl_2_, 0.5 MgCl_2_, 10 HEPES, 5.5 glucose, 0.01 A23187 pH 7.4. Minimal Fluo-4- AM or Rhod-2- AM fluorescence (F_min_) was measured during exposure to Ca^2+^-free calibration solution 1 and maximal Fluo-4-AM or Rhod-2- AM fluorescence (F_max_) during exposure to calibration solution 2 containing [Ca^2+^] of 4 mM (Fig. S4). To transform Fluo-4-AM and Rhod-2-AM fluorescence into free [Ca^2+^], the equation first formulated by Grynkiewicz and colleagues was used [76]: [Ca^2+^] = Kd (F − F_min_) / (F_max_ − F) where Kd is the apparent Ca^2+^ dissociation constant of the indicator; F_min_ and F_max_ are the fluorescence intensities at zero and saturating [Ca^2+^], respectively; and F is the fluorescence intensity at any given time [75].

### Analysis of the mitochondrial inner membrane potential (ΔΨ_m_)

The ΔΨ_m_ was evaluated by monitoring the fluorescence of tetramethylrhodamine ethylester (TMRE, Abcam, Cambridge, UK) used in nonquenching mode [26]. Rat primary cortical neurons were grown on poly-D-lysine-coated glass coverslips and then subjected to the specific experimental protocol. After a loading phase with 10 nM TMRE in the culture medium at 37 °C for 30 min, neurons were washed twice with PBS and then permeabilized for 10 min with digitonin 5 µM, in intracellular buffer with the following composition (in mM): 135 KCl, 10 NaCl, 20 HEPES, 5 pyruvate, 2 glutamate, 2 malate, 0.5 KH_2_PO_4_, 1 MgCl_2_, 5 EGTA, and 1.86 CaCl_2_. Digitonin at low concentrations selectively renders the plasma membrane permeable without affecting the integrity of cellular organelles, such as mitochondria [77]. After permeabilization, neurons were continuously perfused with the intracellular solution containing TMRE (10 nM) and digitonin (5 µM). In these experimental conditions, the TMRE fluorescence decreases with mitochondrial membrane depolarization [26]. Images were acquired using a 510 LSM microscope (Carl Zeiss), with TMRE fluorescence (543 nm excitation/580–700 nm emission) collected every 5 s. The basal levels of the ΔΨ_m_ were monitored for ~300 s. As an internal control, 20 µM of carbonyl cyanide ptrifluoromethoxyphenylhydrazone (FCCP) was added after 180 s. Analysis of the fluorescence intensity was performed offline after image acquisition.

### Evaluation of intracellular ATP levels

A commercially available luciferase-luciferin system (ATPlite, Perkin Elmer) was used to measure the intracellular ATP levels, as previously described [25, 26, 73]. Briefly, SH-SY5Y cells were previously differentiated on 96-well ViewPlate (Perkin Elmer) and then exposed to the indicated treatments in DMEM medium. Primary rat cortical neurons were plated on 12 multiwell plates, then treated in Neurobasal medium according to assigned experimental groups, and finally lysed and transferred to a 96-well ViewPlate (Perkin Elmer) for the ATP assay. The intracellular ATP levels were analyzed with a luminescence counter (Victor Multilabel Counter, Perkin Elmer), normalized to the respective protein content, and expressed as percentages of the control value.

### Immunocytochemistry

#### Primary antibodies

Aβ_1-42_ protein was identified by using a mouse monoclonal IgG1 antibody (clone 12F4, Cat. 805501, Biolegend, San Diego, CA, USA, dilution 1:100 in PBS with 1% BSA). As for pTau protein, a human PHF-Tau monoclonal IgG antibody was used (clone AT100, Cat. MN1060, recognizing Thr212 and Ser214, Thermo Scientific, Milano, Italy, dilution 1:1000 in PBS with 1% BSA).

#### Immunofluorescence staining

After the experimental procedures, the cells were firstly loaded with MitoTracker 300 nM (MitoTracker Red CMXRos M7512 Invitrogen) [25, 73] for 30 min at 37 °C, then fixed with PBS and 3.7% formaldehyde for 30 min at RT and finally permeabilized with PBS-Triton X-100 for 5 min at RT. Subsequently, the cells were incubated with the primary antibodies (Aβ_1-42_ or pTau AT100) for 1.5 h at RT. A conjugated secondary antibody Alexa Anti-Mouse 488 (Cat. A11059 Thermo Scientific, dilution 1:200), was used to detect the immunoreactions.

### Western blot analysis

Western blot experiments were conducted on total lysates of rat primary cortical neurons, RA-differentiated SH-SY5Y cells, and mitochondrial proteins-enriched fractions of RA-differentiated SH-SY5Y [78, 79]. Total lysates of rat primary cortical neurons and RA-differentiated SH-SY5Y cells were prepared in B-buffer 1X containing (in mM): 150 NaCl, 10 Tris-HCl (pH 7.4), 1 EDTA (pH 8.0), 1% SDS. Mitochondria and cytosolic fractions were collected from about 8–10 subconfluent (about 80%) 100 mm Petri dishes of RA-differentiated SH-SY5Y cells according to the manufacturer’s instructions of a commercially available kit (QProteome Mitochondria Isolation kit, Qiagen, Milan, Italy) [80]. Protein content was assessed by the Bradford method (Bio-Rad, Milan, Italy). Samples containing equal amounts of protein (20–40 μg) were prepared in 6× Laemmli sample buffer with 2–mercaptoethanol and boiled for 10 min. Proteins were electrophoretically separated onto an 8-15% SDS-polyacrylamide gel and electro-transferred to a nitrocellulose membrane (Bio-Rad). To reduce nonspecific interactions, the membranes were blocked with non-fat dry milk (5% in PBS buffer), for 1 h, at RT. After the blocking phase, the membranes were incubated with the appropriate primary antibody overnight at 4 °C. To detect the immunoreactions, membranes were incubated with the appropriate secondary antibody conjugated to horseradish peroxidase (Goat anti-rabbit IgG-HRP Cat. sc-2004, Donkey anti-goat IgG-HRP Cat. sc-2020, Santa Cruz, CA, USA; Goat anti-Mouse IgG (H + L) HRP Cat. 62-6520, Thermo Scientific) for 1 h at RT. Blots were then developed with an enhanced chemiluminescence detection kit (Super Signal West Femto kit, Thermo Scientific), and images were acquired with a Uvitec Cambridge Chemiluminescence Imaging System (Cambridge, UK).

#### Primary antibodies

Primary antibodies used in this work are listed below: anti-Aβ clone 6E10 (Cat. 803004, dilution 1:500; Biolegend), anti-phospho-mTOR (Cat. sc-293133, dilution 1:1000, Santa Cruz), anti-phospho AMPKα (Cat. 2535, dilution 1:1000, Cell Signaling, Danvers, MA, USA), anti-Adenine Nucleotide Translocator (ANT, Cat. sc-9299, dilution 1:1000, Santa Cruz) and anti-Glyceraldehyde-3-Phosphate Dehydrogenase (GAPDH, Cat. 60004-1-Ig, dilution 1:10,000, Proteintech, Rosemont, IL, USA), anti-β-actin (Cat. sc-47778, dilution 1:1000, Santa Cruz). Band densities were analyzed with Uvitech Nine Alliance analysis software (Cambridge, UK) and normalized to the appropriate housekeeping protein expression.

### Drugs and chemicals

Ret, XE-991, and ICA-27243 were obtained from Sigma, Tocris, and MedChemExpress, respectively. All the remaining chemicals were of analytical grade and were obtained from Sigma.

### Data analysis

Data were reported as the mean ± standard error of the mean (S.E.M.). GraphPad Prism® 5 software (San Diego, USA) was used for the statistical analysis of the results. One-way ANOVA analysis followed by Dunnett’s post hoc test was used to calculate the differences between the mean values; the minimal level of significance chosen was *p* < 0.05.

## Supplementary information

Supplementary information

Fig. S1

Fig. S2

Fig. S3

Fig. S4

Fig. S5

Fig. S6

Fig. S7

## Data Availability

The data generated and/or analyzed during the current study are available from the corresponding author on reasonable request.
