# Supplementary material for: A new K+channel-independent mechanism is involved in the antioxidant effect of XE-991 in an in vitro model of glucose metabolism impairment: implications for Alzheimer’s disease
Source: Cell Death Discov. 2022 Sep 20;8:391. doi: 10.1038/s41420-022-01187-y (PMC9489689; doi:10.1038/s41420-022-01187-y)
Supplement: Supplementary file 1 — Supplementary information [file 41420_2022_1187_MOESM1_ESM.docx]

**Supplementary information**

**Materials and methods**

*RNA extraction, reverse transcription and quantitative PCR*

Total RNA was extracted from SH-SY5Y and RA-differentiated cells using total RNA Purification Kit (Norgen Biotek, Thorold, Canada) and reverse transcribed to cDNA using SensiFAST cDNA Synthesis Kit (Meridian Bioscience, Memphis, USA), according to the manufacturer’s instruction. Quantitative PCR experiments were carried out in a real-time PCR system (7500 fast; Applied Biosystems, Monza, Italy) using the SYBR Green detection technique and specific primers, as previously described (Iannotti et al, JPET 2010). Cycle threshold (Ct) for each gene of interest was calculated using 7500 fast Applied Biosystems SDS Software, normalized to two reference genes, namely glyceraldehyde-3-phosphate dehydrogenase (GAPDH) and the ribosomal RNA gene S16 (S16). Data were expressed using the 2^-ΔCt^ formula. No template controls and reverse transcriptase-negative controls were run alongside all reactions to assess contamination. Mastermix for the qPCR experiments were purchased from Smobio (Hsinchu City, Taiwan).

ND = no RA-differentiation; RA-diff = RA-differentiated.

**Figure legend**

**Figure S1.** mRNA expression of KCNQ channels in SH-SY5Y and RA-differentiated SH-SY5Y cells. Each column represents the mean ± S.E.M. of n = 5 independent experiments.

**Figure S2.** Effect of ICA-27243 against GA-induced cell damage in RA-differentiated SH-SY5Y cells. Cells were exposed to ICA-27243 (30 µM) for 1 h and then treated with GA (1 mM) for 24 h (without removing ICA-27243). In each experiment MTT reduction was expressed as a percentage of the control value. Statistical differences among means were assessed by one-way ANOVA followed by Dunnett’s post hoc test. F (3, 16) = 29.44. Each column represents the mean ± S.E.M. of n = 5 independent experiments performed in triplicate. *Significant versus Ctl and ICA-27243 (p < 0.0001).

**Figure S3.** Effect of 1 h exposure to XE-991 (300 nM) on SOD activity in RA-differentiated SH-SY5Y cells. Cells were exposed to XE-991 (300 nM) for 1 h and then treated with GA (1 mM) for 24 h (without removing XE-991). In each experiment SOD activity was expressed as a percentage of the control. Statistical differences among means were assessed by one-way ANOVA followed by Dunnett’s post hoc test. Each column represents the mean ± S.E.M. of n = 6 independent experiments performed in triplicate. F (2, 15) = 9.073. *Significant versus all groups (p < 0.01); **significant versus GA (p < 0.01).

**Figure S4.** In situ calibration of Fluo 4-AM (A) and Rhod 2-AM (B) in rat cortical neurons. This method of calibrating Ca^2+^-sensitive fluorescent dyes in situ has been described in detail in the “Materials and Methods” section.

**Figure S5.** Western blot images of Aβ clone 6E10, ANT and GAPDH expression in total lysates of wild type and 3xTg mice and in mitochondrial and cytosolic fractions isolated from RA-differentiated SH-SY5Y cells and (see “Results” section).

wt = wild type; 3xTg = triple transgenic.

**Figure S6.** Western blot images of p-AMPK (Thr172) expression in total lysates of both primary rat cortical neurons and RA-differentiated SH-SY5Y cells (see “Results” section).

**Figure S7.** Western blot images of p-mTOR (Ser2448) in expression in total lysates of both primary rat cortical neurons and RA-differentiated SH-SY5Y cells (see “Results” section).
