# Supplementary figures and images for: A new K+channel-independent mechanism is involved in the antioxidant effect of XE-991 in an in vitro model of glucose metabolism impairment: implications for Alzheimer’s disease
Source: Cell Death Discov. 2022 Sep 20;8:391. doi: 10.1038/s41420-022-01187-y (PMC9489689; doi:10.1038/s41420-022-01187-y)

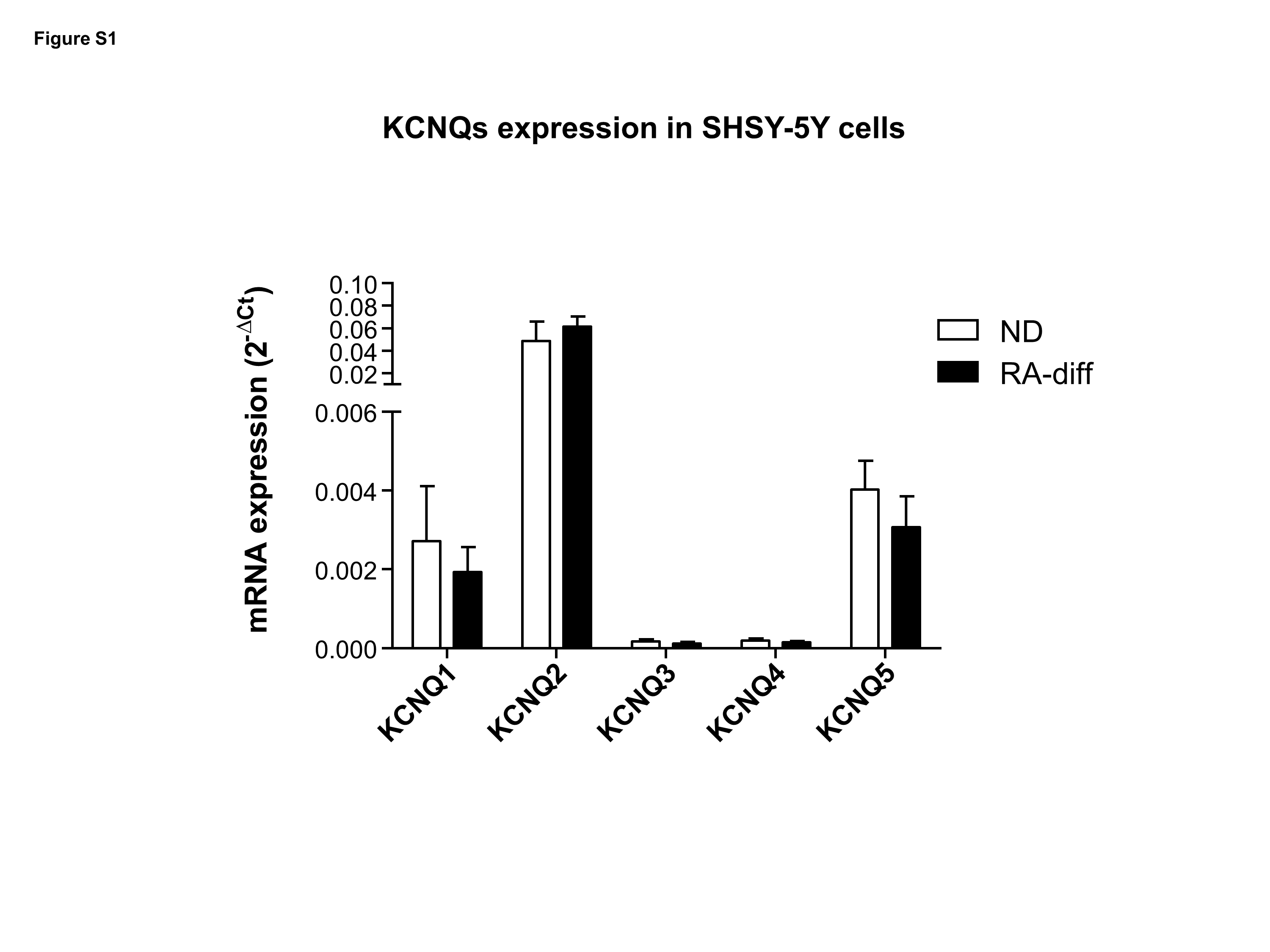

Supplement: Supplementary file 2 — Fig. S1 [file 41420_2022_1187_MOESM2_ESM.tif]

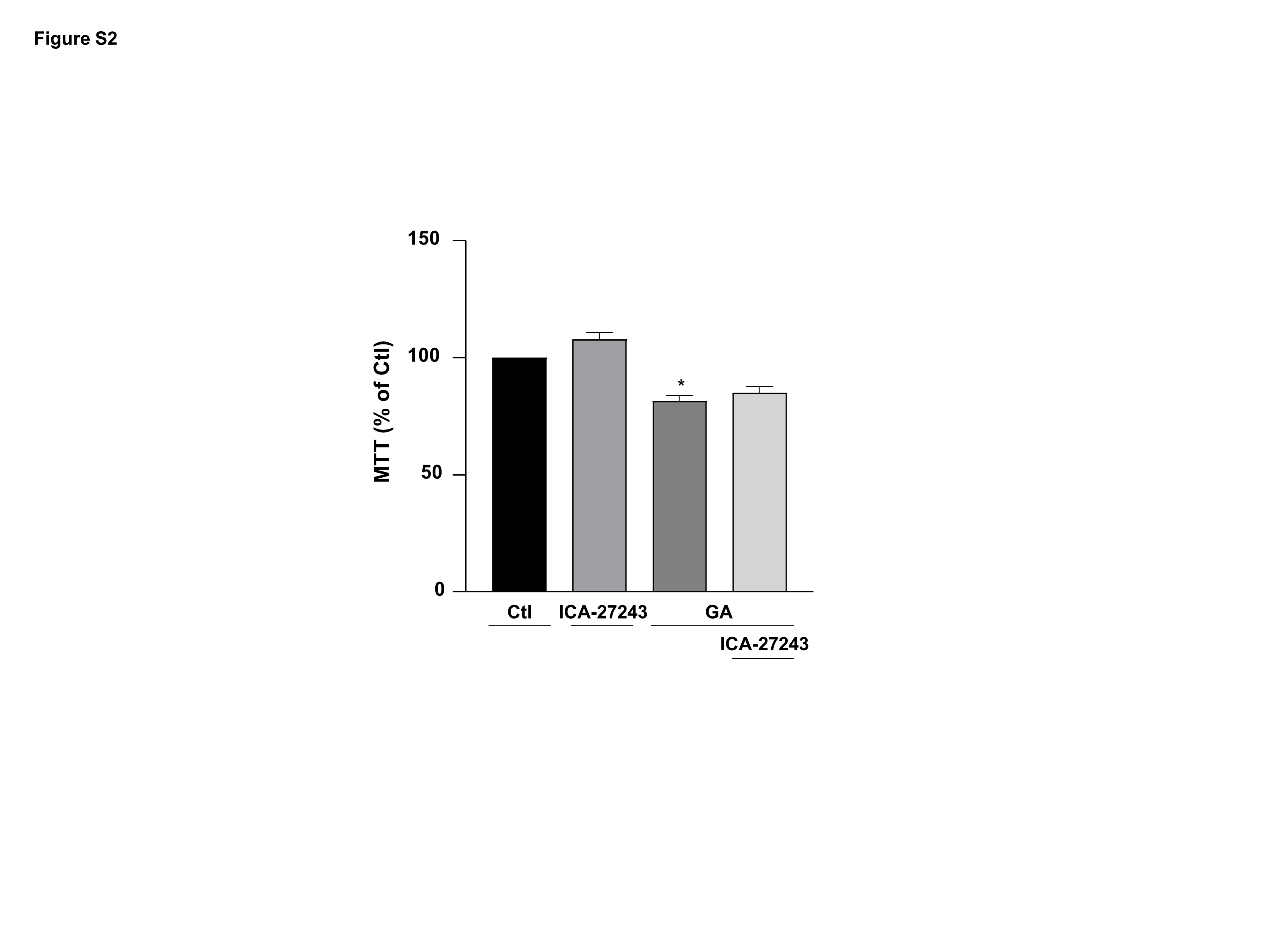

Supplement: Supplementary file 3 — Fig. S2 [file 41420_2022_1187_MOESM3_ESM.tif]

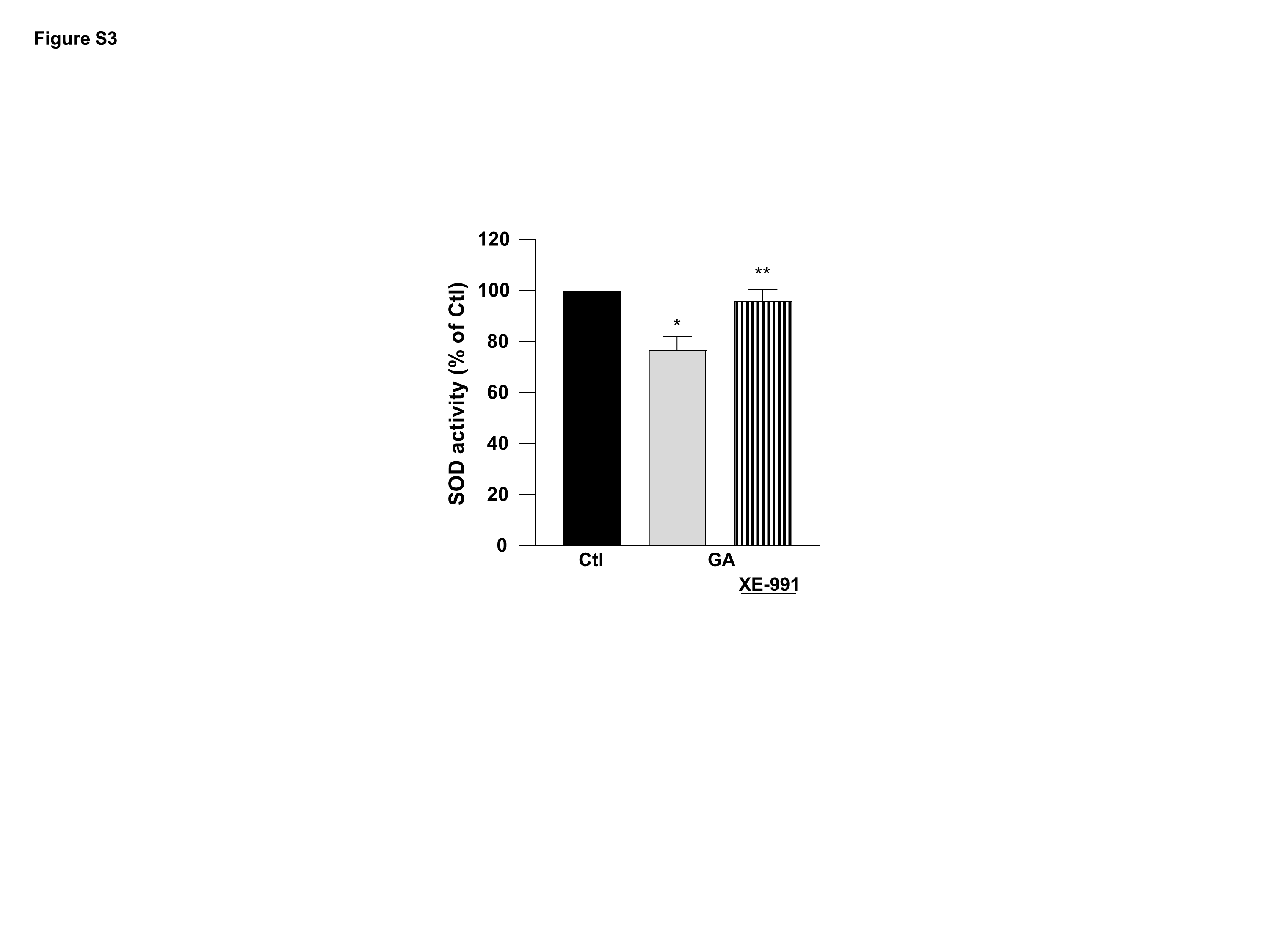

Supplement: Supplementary file 4 — Fig. S3 [file 41420_2022_1187_MOESM4_ESM.tif]

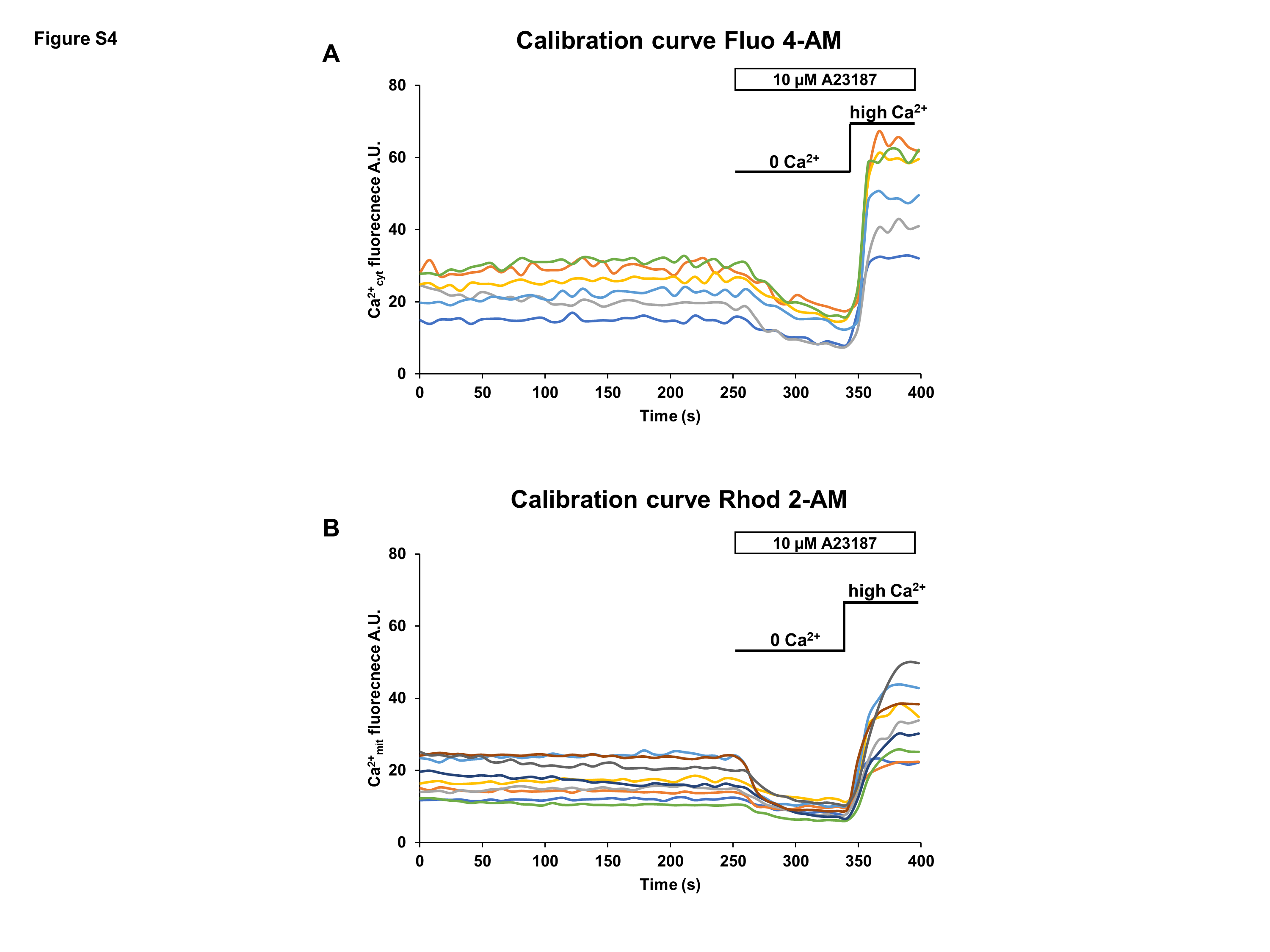

Supplement: Supplementary file 5 — Fig. S4 [file 41420_2022_1187_MOESM5_ESM.tif]

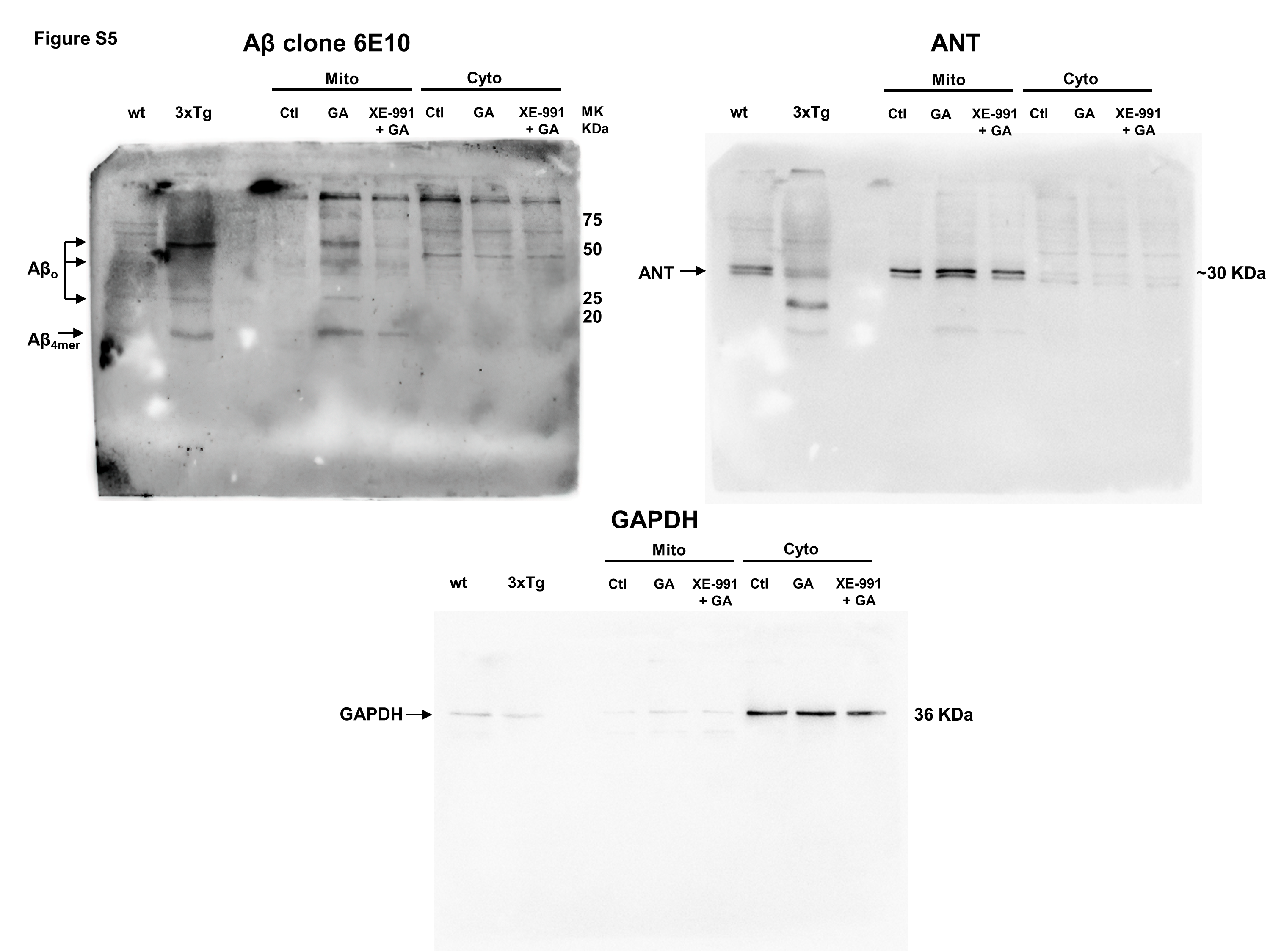

Supplement: Supplementary file 6 — Fig. S5 [file 41420_2022_1187_MOESM6_ESM.tif]

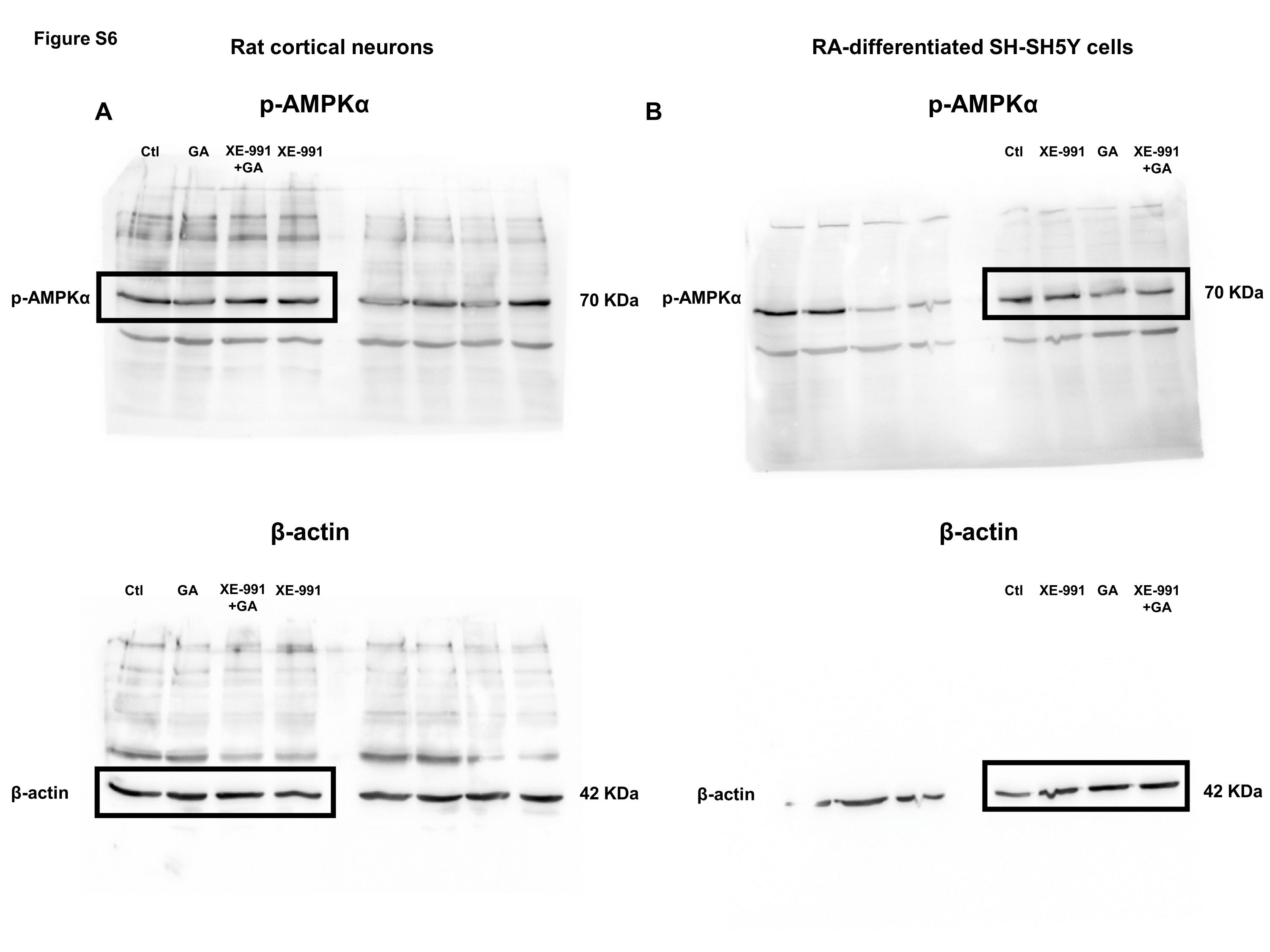

Supplement: Supplementary file 7 — Fig. S6 [file 41420_2022_1187_MOESM7_ESM.tif]

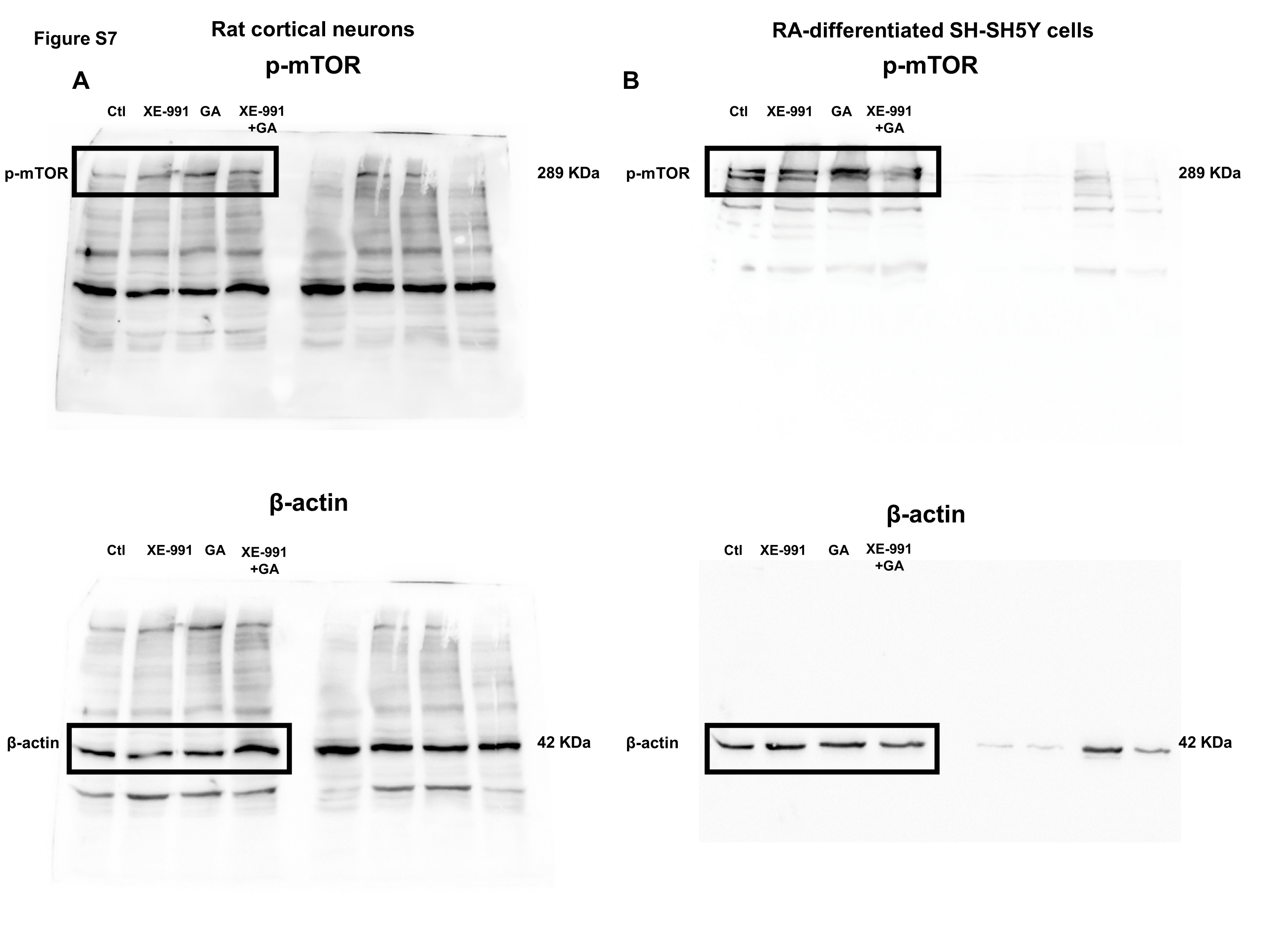

Supplement: Supplementary file 8 — Fig. S7 [file 41420_2022_1187_MOESM8_ESM.tif]
